# A review of the activation triggers and reasons for stand downs of a Helicopter Emergency Medical Service (HEMS)

**DOI:** 10.1186/1757-7241-22-S1-P5

**Published:** 2014-07-07

**Authors:** Elizabeth G Evans, Anthony Hudson, Emily McWhirter, Richard Lyon

**Affiliations:** 1 St George’s University of London, UK; 2 Kent, Surrey, Sussex Ambulance Trust (KSSAAT) - Helicopter Emergency Medical Service, UK

## Background

HEMS crews – a pilot, doctor and paramedic – provide advanced pre-hospital care to acutely ill or injured patients. Sometimes HEMS is deployed only to be no longer required; a stand down. This means a vital resource is unavailable whilst costing the charity money and requiring crews to take unnecessary risks.

## Method

Data was collected from the KSSAAT database – HEMSbase. For each stand down job (June 2013 – August 2013) the mechanisms of injury, reasons for stand downs, times of stand downs and call dispatch types were noted. Monthly summary data sheets were then used to compare this data to that of successful jobs.

## Results

Of the 561 jobs HEMS was deployed on in this time period, 226 (38%) were stand downs. All mechanisms of injury has a >25% stand down rate with assault jobs having the most (57%). 34% of stand downs were due to major injuries turning out to be minor although 20% of jobs did not have documented reasons. Interestingly, only 10% of stand downs were due to technical issues such as weather, re-tasking (sent to another job) or delays en route. In total, 61% of stand downs were immediate dispatches (HEMS is deployed on initial information received) compared to the 30% that were sent after interrogation (more information is gathered). Most stand downs occurred at night (19:01 - 07:00) and there were peaks at paramedic shift change overs (07:00 - 19:00).

## Conclusion

Improving the quality of initial information could improve tasking accuracy and hence reduce stand downs. The improvements are two-fold with more tasking training for staff and development of the dispatch criteria.
